# Safety and Tolerability of SGLT2 Inhibitors in Cardiac Amyloidosis—A Clinical Feasibility Study

**DOI:** 10.3390/jcm13010283

**Published:** 2024-01-04

**Authors:** Maximilian J. Steinhardt, Vladimir Cejka, Mengmeng Chen, Sabrina Bäuerlein, Julia Schäfer, Ali Adrah, Sandra M. Ihne-Schubert, Aikaterini Papagianni, K. Martin Kortüm, Caroline Morbach, Stefan Störk

**Affiliations:** 1 Interdisciplinary Amyloidosis Center of Northern Bavaria, University Hospital Würzburg, 97080 Würzburg, Germanyihne_s@ukw.de (S.M.I.-S.); kortuem_m@ukw.de (K.M.K.); stoerk_s@ukw.de (S.S.); 2 Department of Internal Medicine II, University Hospital Würzburg, 97080 Würzburg, Germany; 3 Department of Internal Medicine I, University Hospital Würzburg, 97080 Würzburg, Germany; chen_m2@ukw.de (M.C.);; 4 CIRCLE—Centre for Innovation Research, University Lund, 22362 Lund, Sweden; 5 Department of Internal Medicine IV, University Hospital Gießen, 35392 Gießen, Germany; 6 Department of Neurology, University Hospital Würzburg, 97080 Würzburg, Germany; 7 Department Clinical Research & Epidemiology, Comprehensive Heart Failure Center, University Hospital Würzburg, 97080 Würzburg, Germany

**Keywords:** heart failure, chronic kidney disease, amyloidosis, SGLT2 inhibitors

## Abstract

Sodium-glucose transport protein 2 inhibitors (SGLT2i) slow the progression of renal dysfunction and improve the prognosis of patients with heart failure. Amyloidosis constitutes an important subgroup for which evidence is lacking. Amyloidotic fibrils originating from misfolded transthyretin and light chains are the causal agents in ATTR and AL amyloidosis. In these most frequent subtypes, cardiac involvement is the most common organ manifestation. Because cardiac and renal function frequently deteriorate over time, even under best available treatment, SGLT2i emerge as a promising treatment option due to their reno- and cardioprotective properties. We retrospectively analyzed patients with cardiac amyloidosis, who received either dapagliflozin or empagliflozin. Out of 79 patients, 5.1% had urinary tract infections; 2 stopped SGLT2i therapy; and 2.5% died unrelated to the intake of SGLT2i. No genital mycotic infections were observed. As expected, a slight drop in the glomerular filtration rate was noted, while the NYHA functional status, cardiac and hepatic function, as well as the 6 min walk distance remained stable over time. These data provide a rationale for the use of SGLT2i in patients with amyloidosis and concomitant cardiac or renal dysfunction. Prospective randomized data are desired to confirm safety and to prove efficacy in this increasingly important group of patients.

## 1. Introduction

The sodium–glucose transport protein-2 (SGLT2) is a co-transporter located in early proximal tubule. It is responsible for the reabsorption of over 90% of the filtered glucose in a sodium-dependent manner [1]. The plasma glucose-lowering effects of SGLT2 inhibitors (SGLT2i) have been known since the mid-1990s. In 2014, dapagliflozin and empagliflozin were approved for the treatment of type-2 diabetes mellitus.

Subsequently, to prove their efficacy in patients with heart failure, dapagliflozin was studied in the DAPA-HF and DELIVER trials, and empagliflozin was studied in the EMPEROR-Reduced and EMPEROR-Preserved trials [2,3,4,5]. The totality of evidence from these landmark studies suggests that, in patients with heart failure, SGLT2i reduce the risk of cardiovascular death and heart-failure-related hospitalizations regardless of the underlying left-ventricular function and of the presence or absence of diabetes mellitus [4,6,7,8]. Although not yet fully understood, these profound clinical benefits may be the consequence of favorable effects on mitochondrial function, inflammatory pathways, and rhythmogenesis [9,10,11].

The evidence for the efficacy of SGLT2i in chronic kidney disease (CKD) is also strong. SGLT2i were reported to reduce the glomerular filtration pressure and thereby mitigate the long-term decline in the estimated glomerular filtration rate (eGFR) and albuminuria [12]. Clinically, SGLT2i were found to reduce the risk of hospitalization [13]. Accordingly, the use of SGLT2i in patients with CKD is recommended to slow the decline in renal function by the Kidney Disease: Improving Global Outcomes Organization (KDIGO), regardless of the presence or absence of diabetes mellitus [14].

However, the application of SGLT2i in clinical routine still has its restrictions and may exert side effects. Prior to prescription, an adequate assessment of kidney function is required as the clinical effects of SGLT2i in severe CKD (i.e., glomerular filtration rate below 20 mL/min/1.73 m^2^ or end stage renal disease) are still under investigation. Because glucose transport in urine is driven by sodium reabsorption, the inhibition of SGLT2 also increases sodium loss and can cause a moderate reduction in systolic blood pressure and subsequently postural symptoms [15]. Because of the osmotic effect of increased urine sodium and glucose, SGLT2i also exert a mild diuretic effect. Thus, SGLT2i are not recommended in patients with low blood pressure or pre-existing hyponatremia. Furthermore, patients with SGLT2i medication may be at higher risk for urinary tract infections (UTIs) and genital mycotic infections, most likely due to increased urine glucose concentration [16].

Due to their overall positive effects, the Food and Drug Administration and European Medicines Agency approved the use of empagliflozin and dapagliflozin in heart failure (HF) with preserved (HFpEF), mid-range (HFmrEF), and reduced (HFrEF) as well as CKD. Still, there remains a knowledge gap for specific causes of HF, which were either excluded from the aforementioned trials or unavailable for subgroup analyses.

Amyloidosis is caused by the deposition of insoluble abnormal protein aggregates that adversely affect normal organ function. Amyloidotic fibrils originating from misfolded transthyretin and light chains are the causal agents in the most frequent subtypes, i.e., ATTR and AL amyloidosis. While organ involvement is determined by the attributes of the precursor protein, cardiac involvement defines ATTR amyloidosis and is the most common manifestation in AL, constituting 70–80%. At the time of initial diagnosis, most patients already show symptoms of heart failure.

Moreover, renal involvement is caused by glomerular amyloidotic deposition with subsequent albumin-predominant proteinuria, and tubular atrophy with interstitial fibrosis. About 50–60% of AL cases exhibit amyloid deposits, while kidney involvement in ATTR has only been described in hereditary cases [17]. Survival and quality of life in amyloidosis are dependent on the long-term functionality of the involved organs. Because cardiac and renal function frequently deteriorate over time in these patients, even under the best available treatment, SGLT2i emerge as a promising treatment option for this patient population.

To address this knowledge gap, we retrospectively analyzed patients with amyloidosis, who received either dapagliflozin or empagliflozin as part of their routine clinical treatment and investigated the potential side effects as well as the course of cardiac, renal, hepatic, and hematologic function parameters.

## 2. Patients and Methods

In the Interdisciplinary Amyloidosis Center located at the University Hospital Würzburg, patients with suspected amyloidosis and all subtypes of established amyloidosis are routinely subjected to a comprehensive and standardized diagnostic work-up. Evaluations include genetic testing, serologic and hematologic parameters, urine, echocardiography, quality of life assessment, and physical performance testing. In particular, the type and severity of cardiac involvement, the presence of diabetes mellitus, and the degree of kidney dysfunction are recorded. Every three to six months, patients present for follow-up and are routinely asked for signs of infection, hypotension, and the other potential side effects of current medications, and are evaluated for identical parameters. In these patients, based on the considerations described above and once cardiac compensation had been reached, SGLT2i were initiated upon clinical indication.

For the current analysis, we retrospectively identified patients who had received treatment with SGLT2i at our center. To assess safety and outcomes in these patients, we assessed the respective longest evaluable time interval for SGLT2i medication and evaluated all the laboratory markers available at both initiation and last presentation, including cardiac, renal, hepatic, and hematologic parameters. We also assessed the respective iterative measurements of 12-lead electrocardiography, 6 min walk distance, medication, and the structured physical examination including clinical signs and symptoms of heart failure such as edema, nocturia, and NYHA functional status.

Echocardiography was performed according to a pre-specified standard including parasternal, apical, and subcostal views using a Vivid E9 or Vivid E95 machine (GE Healthcare, Horten, Norway). All dimensions, functional systolic/diastolic parameters, and valves were assessed and included the recording of left-ventricular-outflow tract diameter from the parasternal long axis and pulsed-wave Doppler in an apical five-chamber view to calculate the cardiac output and apical four-, two-, and three-chamber views to determine the left-ventricular-ejection fraction in the Simpson biplane method and global longitudinal strain. Renal function was estimated via the Chronic Kidney Disease Epidemiology Collaboration estimator (CKD-EPI).

We tested for differences of normally distributed variables with the paired *t*-test. For the comparison of matched samples without normal distribution, we used the paired Wilcoxon signed-rank test. Differences between the categories of the functional NYHA class were analyzed with Pearson’s chi-squared test. A *p*-value of 5% was considered statistically significant. No adjustment for multiple testing was performed, as all analyses were be considered exploratory. The latest data included into the dataset were collected in 30 July 2023. The Ethics Committee of the Medical Faculty of Würzburg conceded to the conduct of the study outside of a formal ethical consent (waiver # 20230208 03).

## 3. Results

We identified 79 patients with amyloidosis, who had received either dapagliflozin or empagliflozin between January 2020 and May 2023 for a duration of at least 3 months. Information of the baseline visit and at least one follow-up visit was available for all. Their median age was 80 years (range 45–89), 62 (79%) were men, 66 (86%) were diagnosed with wild-type ATTR (wtATTR), and 13 (14%) patients had AL amyloidosis. All patients exhibited cardiac involvement, and 11 out the 14 AL patients had renal involvement. All AL patients were in Mayo stage III and had achieved at least a very good partial hematologic and stable organ response prior to commencing SGLT2i.

The median left-ventricular-ejection fraction (LVEF) in our cohort was 53% (quartiles 45; 59). HFpEF was present in 49 patients (65%), HFmrEF in 16 patients (21%), and HFrEF in 11 patients (15%). The initial mean estimated glomerular filtration (eGFR) rate was 50 ± 18.1 mL/min/1.73 m^2^. In general, the cardiac and renal functions of AL and ATTR patients was comparable (Table 1).

Median follow-up was 8.2 months (quartiles 6.8; 13.0). Eight patients (10%) had already received treatment with SGLT2i before their first visit to our center. Six of them (75%) had diabetes. The prevalence of diabetes mellitus in the total sample was 22%. There were no patients qualifying for SGLT2i in whom the substance class could not be initiated due to pre-existing intolerance. All patients with ATTR received a disease-modifying medication. Amongst all patients, loop diuretics were the most frequent co-medication (79%) followed by ACE inhibitors or AT1 blockers (57%; Table 2).

Echocardiography revealed several changes from baseline to the end of the follow-up period. Mean (±SD) E/E’ decreased (−1.4 ± 5.2; *p* = 0.03), while the left-ventricular-end-diastolic volume increased (+8.1 ± 3.9 mL, *p* = 0.04) and cardiac output (−0.5 ± 0.2 L/min, *p* = 0.002) and stroke volume decreased (−6.3 ± 2.2 mL, *p* = 0.006). The right ventricular wall thickness increased by 0.51 ± 0.3 mm (*p* = 0.05) and the maximal tricuspid valve pressure gradient decreased by 3.3 ± 1.0 mmHg (*p* = 0.003). For a detailed analysis of echocardiac parameters, please refer to the supplement (Table S1).

**Figure 1:**
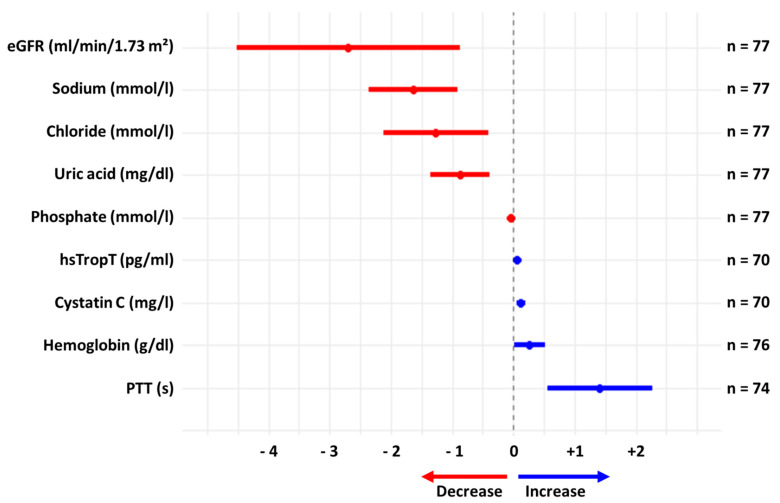
Significant alterations in laboratory markers (absolute changes) in 79 amyloidosis patients after initiation of treatment with SGLT2i. eGFR = estimated glomerular filtration rate. Hs-TropT = high-sensitivity troponin T. PTT = partial thromboplastin time. Hs-TropT was log-transformed (natural logarithm) to approximate for normal distribution. Median follow-up was 8.2 months.

We found a significant decrease in eGFR by 2.7 ± 8.0 mL/min (*p* = 0.006) and a rise in cystatin C by 0.12 ± 0.27 points (*p* < 0.001). There were no episodes of acute kidney injury necessitating the interruption of SGLT2i therapy. Sodium levels decreased by 1.6 ± 3.2 mg/dL (*p* < 0.001), while potassium levels remained unchanged (*p* = 0.36). We observed no sodium or glucose levels below the lower range of normal in our cohort. Other significant alterations included the hemoglobin concentration (+0.26 ± 1.10 mg/dL, *p* = 0.04) and highly sensitive troponin T (+2.7 ± 9.9 pg/mL; +5.9%, *p* = 0.002). Figure 1 gives an overview of the relevant laboratory changes. All analyzed parameters can be found in the supplement (Table S2).

Four patients (5%) reported urinary tract infections; two of them subsequently stopped the medication for this reason. The other two patients proceeded without further complications. There were no events of severe infectious complications. Two patients died due to unrelated complications (one hip joint prosthesis infection and one cardiac decompensation without infection). Importantly, hepatic markers (alanine transaminase, aspartate transaminase, gamma-glutamyltransferase, and bilirubin) and NT-proBNP remained unchanged. We found no significant increase in the inflammatory parameters, serum leukocytes, or bacteriuria. These observations were similar for patients with ATTR or AL. All analyzed parameters can be found in the supplement (Table S2).

**Figure 2:**
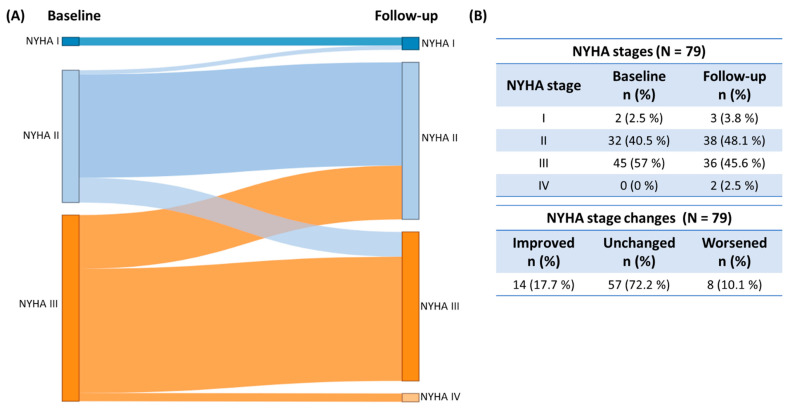
(**A**) Visualization of (**B**) numeric changes in the NYHA functional class from primary presentation to follow-up. Change in the NYHA functional status (*p* < 0.001) at primary presentation and follow-up. Median follow-up was 8.2 months. Patients at risk: n = 79. NYHA: New York Heart Association functional class.

There were no incident cases exhibiting hypotension or nausea or atrial fibrillation. Furthermore, no worsening of leg edema or nocturia was observed. The mean 6 min walk distance measured for both time points in 56 patients remained unchanged over the follow-up period (−9.9 ± 67.2 m, *p* = 0.27), while NYHA functional class improved significantly (*p* < 0.001) as displayed in Figure 2.

## 4. Discussion

The current retrospective analysis found that treating consecutive patients with amyloidosis for a median duration of 8 months with SGLT2i was not associated with major or unexpected adverse effects. In fact, the spectrum of side effects and the frequency of drug discontinuation were lower than those previously reported in large trials [2,6]. Although amyloidosis expectedly was associated with hypotension and orthostatic problems, we did not observe an aggravation of these states after initiating SGLT2i therapy. Furthermore, the assessed changes in biomarkers and echocardiography parameters pointed towards a more favorable volume status under SGLT2i treatment. Nevertheless, prospective randomized data are needed to confirm the safety and prove efficacy in this particular fragile group of patients.

SGLT2i are approved for their use in patients with HF and CKD, which also are common manifestations of systemic amyloidosis. This provides a rationale for the use of SGLT2i in ATTR and AL amyloidosis. Nevertheless, patients with amyloidosis were excluded from large outcome trials and there is a lack of knowledge regarding the safety and efficacy of SGLT2i in this special patient collective. Dobner et al. only recently reported on a cohort of 34 patients with ATTR cardiomyopathy receiving the SGLT2i dapagliflozin. Three-month follow-up showed that dapagliflozin was well tolerated. Further results pointed towards a more favorable hydration status under dapagliflozin. Our study confirms the safe application of SGLT2i in patients with cardiac ATTR amyloidosis contributing to a potentially more favorable hydration status and extend these results (1) to both SGLT2i available in Germany; (2) to patients with AL amyloidosis; (3) by an extended safety analysis for clinical and laboratory parameters; as well as (4) to a longer observation period [18].

The current study confirms that patients with amyloidosis tolerate SGLT2i well: 5% reported typical associated adverse events, and all of them were UTIs. Only two patients (2.5%) permanently stopped therapy, highlighting the good tolerability for amyloidosis patients as previously described [18]. Real-world and major trial data suggest that UTIs may be rather associated with pre-existent diabetes than SGLT2i medication. Despite the undoubtedly increased risk for genital mycotic infections, we observed none in our study, which overlooked 796 patient–months of SLGT2i treatment. However, because our cohort constituted a selected collective, this may be an underestimation of the true risk associated with SGLT2i pharmacotherapy. In a larger retrospective analysis from Thailand investigating diabetic persons, UTIs occurred after a mean of 16 months after starting SGLT2i therapy [19].

We observed a slight decrease in the glomerular filtration rate by 2.5 mL/min, while cystatin C increased accordingly. These observations are comparable with the effects described in large prospective trials including the EMPA-KIDNEY trial, where after six weeks of treatment, the eGFR decreased by a mean of 3 mL/min/1.73 m^2^. Of note, long-term treatment with empagliflozin for a period of 3 years was associated with a slower decrease in eGFR compared to the placebo—underscoring its nephroprotective actions [20].

Due to the mechanism of action of SGLT2i, we observed an expected drop in sodium levels, while serum glucose levels remain unchanged. Potassium and other electrolytes also remained unaltered, highlighting the safety of SGLT2i use in AL and ATTR amyloidosis. Thus, in general, patients with ATTR and AL amyloidosis tolerated SGLT2i very well.

We found a small but significant increase in hemoglobin under SGLT2i treatment. This may be due to the hemoconcentration that followed the osmotic diuretic effect of SGLT2i and subsequently improved volume management. Consistent with this assumption and as reported by large randomized trials, E/E’, a marker of left-ventricular filling pressure, decreased as did the pulmonary artery pressure [2,4,6,7].

At the same time, amyloidosis is a progressive condition, even under the best available treatment. In this regard, we found an increase in high-sensitivity troponin, right-ventricular-wall thickness, and a decrease in the left-ventricular-stroke volume and cardiac output, which were likely due to the natural course of gradual cardiac worsening in cardiac amyloidosis.

Hence, due to the observations detailed above, pointing towards the progression of amyloid cardiomyopathy, the improvements in hydration status as suggested by favorable changes in respective measures seem unlikely to be related to Tafamidis. This also can be deducted from the pivotal trials and suggested by similar trends in AL and ATTR. Nevertheless, the efficacy of SGLT2i therapy in cardiac amyloidosis has to be proven in larger and prospective trials.

Overall, our real-world findings in patients with both ATTR and AL amyloidosis point towards a favorable safety profile of SGLT2i. Due to the strong evidence in heart failure, SGLT2i medication should be considered in patients with cardiac and renal amyloidosis. The significant changes in echocardiographic and laboratory parameters point towards a more favorable volume status following the treatment with SGLT2i in both patients with AL or ATTR amyloidosis. Proteinuria remained unchanged at the current median follow-up of 8 months, so that a benefit regarding renal function in this population, while likely, remains to be evaluated.

Our study has some limitations, which have to be considered when interpreting the results: we investigated a convenience sample with a modest sample size and the follow-up period is relatively short. The retrospective nature of this unblinded approach as well as the lack of a control group prevent the evaluation of treatment efficacy. Nevertheless, we were able to analyze a well-characterized sample of patients with a rare disease, enabling us to describe the course over time and to evaluate the safety of SGLT2i treatment.

## 5. Conclusions

This retrospective analysis indicated that treatment with SGLT2i in patients with AL or ATTR amyloidosis is safe, well tolerated, and associated with laboratory and echocardiographic alterations compatible with their well-described reno- and cardioprotective effects. SGLT2i treatment is strongly recommended in chronic heart failure and chronic kidney disease and should therefore be considered on an individual basis in patients with amyloidosis.

## Figures and Tables

**Table 1:** Patient baseline characteristics.

	All Patients (n = 79)	Patients with ATTR (n = 65)	Patients with AL (n = 14)
N (% of total cohort)	79 (100)	65 (82.3)	14 (17.7)
Age (years), median (quartiles)	80	80 (75; 84)	64 (59; 68)
Female sex, n (%)	22 (21.5)	13 (32.9)	9 (35.7)
NYHA functional class, median (range)	3 (1–3)	3 (1–3)	2 (1–3)
HFrEF/HFmrEF/HFpEF, n (%)	12 (63)/17 (22)/50 (15)	12 (18)/14 (22)/39 (60)	0 (0)/3 (21)/11 (79)
NT-proBNP (pg/mL), median (quartiles)	2213 (948; 3611)	2159 (1062; 3636)	2387 (617; 3172)
hs-TropT (pg/mL), median (quartiles)	41.3 (29.3; 72.3)	43.9 (29.8; 72.4)	34.3 (13.5; 59.1)
CKD G1/2/3a/3b/4, n (%)	2 (2.5)/22 (27.8)/16 (20.3)/27 (34.2)/11 (13.9)	1 (1.5)/20 (30.8)/11 (16.9)/24 (36.9)/9 (13.8)	1 (7.1)/2 (14.3)/5 (35.7)/4 (28.6)/2 (14.3)
Diabetes, n (%)	17 (21.5)	12 (18.5)	5 (35.7)
Known coronary heart disease, n (%)	23 (29.1)	22 (33.8)	1 (7.1)
Hypertension, n (%)	50 (63.3)	42 (64.6)	8 (57.1)
Atrial fibrillation, n (%)	41 (51.9)	40 (61.5)	1 (7.1)
Pacemaker, n (%)	12 (15.2)	11 (16.9)	1 (7.1)
ICD, n (%)	2 (2.5)	2 (2.5)	0 (0)
CRT, n (%)	2 (2.5)	2 (2.5)	0 (0)

CKD G: Chronic Kidney Disease Grade (KDIGO staging); CRT: cardiac resynchronization therapy, biventricular pacemaker. HFrEF: heart failure with reduced ejection fraction. HFmrEF: heart failure with mid-range ejection fraction. HFpEF: heart failure with preserved ejection fraction. Hs-TropT: highly sensitive troponin T. ICD: implantable cardioverter defibrillator. NT-proBNP: N-terminal pro-hormone brain-natriuretic peptide. NYHA: New York Heart Association.

**Table 2:** Concomitant medication.

	All Patients (n = 79)	Patients with ATTR (n = 65)	Patients with AL (n = 14)
Loop diuretic, n (%)	62 (78.5)	53 (81.5)	9 (64.3)
ACE inhibitor/AT1 antagonist, n (%)	45 (57.0)	32 (52.3)	11 (78.6)
Thiazide diuretic, n (%)	3 (3.8)	3 (4.6)	0 (0)
Sacubitril/valsartan, n (%)	9 (11.4)	7 (10.8)	2 (14.3)
Betablocker, n (%)	41 (51.9)	35 (53.8)	6 (42.9)
Mineralocorticoid receptor antagonist, n (%)	26 (32.9)	25 (38.5)	1 (7.1)
Cardiac glycoside, n (%)	2 (2.5)	2 (3.1)	0 (0)
Amiodarone, n (%)	7 (8.9)	7 (10.8)	0 (0)
Tafamidis, n (%)	n/a	60 (92.3)	n/a

ACE: angiotensin-converting enzyme. AT1: angiotensin II receptor subtype 1. N/a: Not applicable.

## Data Availability

All data, if not given in this article, are available upon request.

## Supplementary Materials

The following supporting information can be downloaded at: https://www.mdpi.com/article/10.3390/jcm13010283/s1, Table S1: Changes in echocardiac parameters during SGLT2i medication. Table S2: Changes in laboratory parameters during SGLT2i medication.
