# Supplementary material for: Safety and Tolerability of SGLT2 Inhibitors in Cardiac Amyloidosis—A Clinical Feasibility Study
Source: J Clin Med. 2024 Jan 4;13(1):283. doi: 10.3390/jcm13010283 (PMC10780141; doi:10.3390/jcm13010283)
Supplement: Supplementary file 1 [file jcm-13-00283-s001.zip › jcm-2735493-supplementary.pdf]

Journal of
Clinical Medicine

MDPI

Supplement

Table S1. Changes in echocardiographic parameters during SGLT2i medication.

Value	N	Initial value	Mean change	P-value
**IVSd (mm)**	76	17.6	-0.0	0.87
**LVPWd (mm)**	76	14.2	+0.0	0.13
**RV wall thickness (mm)**	62	6.68	+0.52	**0.05**
**LVEDV biplane (ml)**	73	95.7	+8.14	**0.04**
**LV stroke volume (ml)**	70	75.8	-6.25	**0.01**
**Cardiac output (l/min)**	69	5.10	-0.50	**<0.01**
**LVEF biplane (%)**	76	51.4	-0.29	0.78
**GLPS**	67	-11.9	+0.97	0.55
**MV E/E′ mean**	68	17.6	-1.40	**0.03**
**MAPSE septal (mm)**	70	7.36	-0.46	0.10
**MAPSE lateral (mm)**	70	8.73	-0.31	0.32
**MV E max (ms)**	70	0.87	-0.03	0.07
**MV E′ septal (cm/s)**	70	4.35	+0.01	0.93
**MV E′ mean (cm/s)**	67	5.54	-0.17	0.42
**TAPSE (cm)**	74	1.83	-0.27	0.14
**TV S′ (cm/s)**	63	10.1	+0.06	0.85
**TR max PG (mmHg)**	67	35.4	-3.29	**<0.01**

Table S1: Changes in echocardiographic parameters during SGLT2i medication (all normally distributed). E: early mitral inflow velocity. E′: early LV relaxation velocity. GLPS: global longitudinal peak strain. IVSd: interventricular septal wall diameter. LV: left ventricle. LVEDV: left-ventricular-end-diastolic volume. LVEF: left-ventricular-ejection fraction. LVPWd: left-ventricular posterior wall diameter. MAPSE: mitral annular plane systolic excursion. MV: mitral valve. RV: right ventricle. RVS: lateral tricuspid annulus peak systolic velocity. TAPSE: tricuspid annulus peak systolic excursion. TR max PG: maximal systolic pressure gradient across tricuspid valve. TV-S′: peak systolic tissue velocity. We tested for significant differences in the normally distributed variables with the paired t-test.

Table S2. Changes in laboratory parameters during SGLT2i medication.

A) Normally distributed serological parameters	N	Initial mean	Mean change	Two-sided p
**Sodium (mmol/L)**	77	140.61	-1.64	**<0.01**
**Potassium (mmol/L)**	77	4.53	-0.05	0.33
**Calcium (mmol/L)**	77	2.34	+0.03	0.07
**Chloride (mmol/L)**	74	101.74	-1.27	<0.01
**Phosphate (mmol/L)**	77	1.19	-0.04	**0.05**
**Serum protein (g/dL)**	77	6.74	+0.09	0.15
**Serum albumin (g/dL)**	77	4.24	+0.01	0.77
**Prealbumin (mg/dL)**	36	27.27	-0.03	0.96
**Total cholesterol (mg/dL)**	74	173.65	-2.88	0.55
**Creatinine/(mg/dL)**	77	1.44	+0.09	0.06
**eGFR (CKD EPI. ml/min/1.73 m²)**	77	49.89	-2.67	**<0.01**
**Cystatin C (mg/L)**	70	1.56	+0.12	**<0.01**
**Urea (mg/dL)**	77	59.30	+2.50	0.46
**Uric acid (mg/dL)**	77	8.04	-0.88	**<0.01**
**ALAT (U/L)**	77	22.84	-1.35	0.15
**ASAT (U/L)**	77	30.15	-1.51	0.08
**Choline esterase (U/L)**	62	6472.19	+78.31	0.49
**GGT (U/L)**	76	109.69	-7.15	0.51
**Bilirubin (mg/dL)**	70	0.78	+0.01	0.68
**PTT (s)**	74	27.23	+1.41	**<0.01**
**INR**	74	1.22	-0.03	0.42
**Thrombocytes/nL**	76	191.50	+7.94	0.19
**Hematocrit (%)**	76	40.23	+0.75	0.07
**Hemoglobin (g/dL)**	76	13.51	+0.26	**0.04**
**Leukocytes/nL**	76	7.15	+0.06	0.80

B) Non-normally distributed serological parameters	n	Before initiation median (quartiles)	After initiation median (quartiles)	p-value
**NTproBNP (pg/mL)**	69	2213 (933; 3661.5)	2484 (987; 4551)	0.534
**C-reactive protein (mg/dL)**	48	0.35 (0.17; 0.55)	0.41 (0.25; 0.94)	0.232
**hsTroponinT (pg/mL)**	70	41.3 (28.33; 72.43)	47.95 (28.38; 64.7)	**<0.01**

C) Urinanalysis	n	Before initiation median (quartiles)	After initiation median (quartiles)	p-value
**Erythrocytes (1/µL urine)**	64	3 (1; 5)	2 (1; 4)	0.043
**Leukocytes (1/µL urine)**	63	3 (1; 8.25)	1.5 (1; 7)	0.443
**Bacteria (1/µL urine)**	61	4 (1; 24)	5 (1; 20)	0.737
**Albumin/creatinine (mg/g in urine)**	48	49.45 (21.6; 214.98)	51.5 (20.5; 234.65)	0.532
**Protein/creatinine (mg/g in urine)**	48	143 (100; 441)	155 (109; 350)	0.712

Table S2: Changes in A) normally distributed; B) non-normally distributed variables in blood serum; and C) parameters in urine (all non-normally distributed) during SGLT2i medication. ALAT: alanine transaminase. ASAT: aspartate transaminase. CKD EPI: Chronic Kidney Disease Epidemiology Collaboration estimator. eGFR: estimated glomerular filtration rate. GGT: gamma-glutamyl-transferase. Hs-TropT: highly sensitive troponinT. INR: international normalized ratio for quick values. NT-proBNP: N-terminal pro-brain-natriuretic peptide. PTT: partial thromboplastin time. A) Paired t-test or Wilcoxon signed-rank test was used, according to the nature of the data. B, C) Descriptives of these non-normally distributed variables are reported for before and after the initiation of SGLT2-inhibitor therapy. Pairwise comparisons were analyzed with the Wilcoxon paired signed rank test.
